# Long-Term Outcomes and Surgical Conversion After Immunotherapy in Microsatellite Instability-H Biliary Tract Cancers

**DOI:** 10.1200/PO-25-01156

**Published:** 2026-07-10

**Authors:** Priyanshi Shah, Anina Peersen, Vaibhav Sahai, Amit Mahipal, Nikolas Naleid, Mitesh Borad, Tanios Bekaii-Saab, Mohamad Bassam Sonbol, Umair Majeed, Hani M. Babiker, Conor D.J. O'Donnell, Midhun Malla, Garima Gupta, Caitlyn B. Conboy, Lionel Kankeu Fonkoua, Leslie A. Washburn, Thorvardur R. Halfdanarson, Nicole Peterson, Robert R. McWilliams, Ryan M. Carr, Rondell P. Graham, Patrick Starlinger, Rory Smoot, Susanne Warner, Sumera L. Ilyas, Gregory J. Gores, Lewis Roberts, Julie Heimbach, Fang-Shu Ou, Nguyen H. Tran

**Affiliations:** ^1^ Department of Oncology, Mayo Clinic Rochester, MN; ^2^ Division of Clinical Trials and Biostatistics, Mayo Clinic, Rochester, MN; ^3^ Division of Hematology and Oncology, Department of Internal Medicine, University of Michigan, Ann Arbor, MI; ^4^ Department of Oncology, University Hospitals Seidman Cancer Center and Case Western Reserve University, Cleveland, OH; ^5^ Division of Hematology/Oncology, Mayo Clinic, Phoenix, AZ; ^6^ Division of Hematology/Oncology, Mayo Clinic, Jacksonville, FL; ^7^ Division of Hematology and Oncology, University of Alabama, Birmingham, AL; ^8^ Department of Medical Oncology, OSF Healthcare Cancer Institute, Peoria, IL; ^9^ Department of Pathology, Mayo Clinic, Rochester, MN; ^10^ Department of Surgery, Mayo Clinic, Rochester, MN; ^11^ Department of Gastroenterology and Hepatology, Mayo Clinic, Rochester, MN; ^12^ Department of Transplant Surgery, Mayo Clinic, Rochester, MN

## Abstract

**PURPOSE:**

Deficient mismatch repair/microsatellite instability (dMMR, MSI-H) in biliary tract cancers (BTCs) is observed in 1%-5% of tumors. MSI-H across solid tumors has demonstrated durable response to immune checkpoint inhibitors (ICIs) regardless of the anatomic location. Here, we describe clinical outcomes and surgical conversion of MSI-H BTC treated with ICIs.

**Patients and Methods:**

We conducted a multicenter, retrospective analysis of BTC patients with dMMR/MSI-H between 2017 and 2024 at Mayo Clinic, University of Michigan, and University Hospitals Seidman Cancer Center. Outcomes of interest were surgical resection rate, rate of pathologic response, 2-year overall survival (OS; time from initiation of treatment to death/last follow-up), and time to treatment discontinuation (TTD; time from initiation of treatment to cessation because of any cause). Analyses were descriptive.

**RESULTS:**

Thirty patients with MSI-H BTC were identified: 19 with cholangiocarcinoma, three with gallbladder cancer, and 8 with BTC undefined. The median age at diagnosis was 61.5 years (range, 26-86). Twenty-six (86.7%) patients had advanced stage at diagnosis. Twenty-seven patients (90%) received first-line systemic therapy, and 13 (48.1%) continued onto second line. Six patients (23.1%) were restaged to resectable from advanced disease after ICI therapy and underwent resection; all remained recurrence-free at last follow-up. Of these six patients, 3 (50%) achieved a pathologic complete response. Among those receiving first-line therapy, the median TTD was 9.9 months (95% CI, 5.7 to 14.5 months) and the 2-year OS was 73% (95% CI, 56% to 94%).

**CONCLUSION:**

ICI therapy in patients with MSI-H BTC demonstrated durable response and downstaged some BTC to a resectable stage. dMMR/MSI-H should be performed up-front to guide treatment decisions.

## INTRODUCTION

Biliary tract cancers (BTCs) consist of invasive adenocarcinomas of the biliary tree, including the gallbladder. They account for about 3% of all gastrointestinal malignancies, and although rare, their incidence has been steadily rising over the past 10 years.^1^ Treatment of BTC is often palliative since the majority are diagnosed at an advanced stage where curative surgery is not an option.^2^ These malignancies have a poor prognosis, with a poor 5-year overall survival (OS) of only 10%-40% even after surgical resection of tumor.^3-5^

**CONTEXT**

**Key Objective**
To characterize clinical outcomes and surgical conversion rates in patients with deficient mismatch repair/microsatellite instability (MSI-H) biliary tract cancer (BTC) treated with immune checkpoint inhibitors (ICIs).
**Knowledge Generated**
Among 30 patients with MSI-H BTC treated across three centers, patients with unresectable BTC on systemic therapy demonstrated a 2-year overall survival of 73%. Furthermore, among patients who received ICI as part of their treatment profile, 23% converted to curative surgery and 11.5% achieved complete durable response not requiring surgery.
**Relevance**
Up-front molecular testing of the tumor and administration of ICI in this highly aggressive cancer can help achieve durable disease control and cure in a subset of patients with historically poor outcomes.

A recent shift in the treatment paradigm promising better survival to patients with BTC occurred with the advent of precision medicine and ICIs.^6,7^ The TOPAZ-1 trial, which added a PD-L1 antibody, durvalumab, to the first-line standard of care, gemcitabine and cisplatin (GC), demonstrated statistically significant improvement in survival (median OS 12.8 *v* 11.5 months; hazard ratio [HR], 0.80; CI, 0.66 to 0.97) with comparable rates of grade 3 or 4 adverse events.^2^ This led to the approval by the Food and Drug Administration (FDA) of GC plus durvalumab as the first-line treatment for patients with advanced BTC.^8^ Similarly, the KEYNOTE-966 trial comparing GC and pembrolizumab, PD-1 antibody, with GC alone showed significant improvement in OS (median OS 12.7 *v* 10.9 months; HR, 0.83; CI, 0.72 to 0.95), which led to its FDA approval as well for first-line treatment.^9^ Even with these modest improvements, virtually, all patients will progress on first-line therapy.

New developments in comprehensive genomic analysis have revealed that 40%-56% of BTC harbors specific driver genes which can be targeted using molecular targeted therapy.^10,11^ One area of great interest is the mismatch repair (MMR) pathway which is involved in the maintenance of genomic stability, mutation avoidance, and DNA replication fidelity.^12^ Deficiency in these pathways leads to accumulation of mutations at microsatellite sequences, leading to instability in these regions.^13^

Patients with these microsatellite instability high (MSI-H) tumors have experienced prolonged control of disease from ICIs.^14,15^ KEYNOTE-158 demonstrated that MSI-H patients with advanced cholangiocarcinoma achieved an objective response rate (ORR) of 40.9% (9 of 22 patients)—which is substantially higher than the ORR of 26.7% observed in TOPAZ-1 and 29% in KEYNOTE-966—and a remarkable median OS of 24.3 months despite prior treatment(s).^2,9,16^ The role of PD-1 inhibitors in advanced MSI-H solid tumors has been well-established, with the FDA approval of pembrolizumab for patients with previously treated unresectable or metastatic MSI-H solid tumors in March 2023.^17-19^ Yet, there is a paucity of data with regard to their use as first-line therapy for unresectable tumors or in the neoadjuvant setting or even perhaps as an alternative to operative management in BTC.^20^

Our study aims to investigate real-world outcomes of BTC patients with MSI-H treated with ICI. We seek to characterize the treatment pattern, surgical conversion rate, and nonoperative management after treatment with ICI among patients with unresectable/metastatic disease.

## Patients and Methods

### Overview

The Mayo Clinic Institutional Review Board reviewed and approved this study (IRB23-003728). This retrospective, multi-institutional study relied on review of electronic health records of patients with BTC who were diagnosed between July 1, 2013, and May 1, 2024. This study included patients from Mayo Clinic, the University Hospitals Seidman Cancer Center, and the University of Michigan.

### Patient Eligibility

Patient inclusion criteria consisted of the following: (1) Age 18 years or older at cancer diagnosis, (2) pathologically confirmed evidence of cholangiocarcinoma or gallbladder cancer, (3) at least two clinic visits at the respective institution where patients were seen and treated, and (4) clinical documentation and/or molecular reports of MSI-H status by molecular testing or MMR deficiency by immunohistochemistry (IHC).

### Data Acquisition

Automated natural language processing tools, such as Advanced Text Explorer and Electronic Medical Record Search Engine,^21^ were used to search the electronic medical records at Mayo Clinic and the University of Michigan, respectively. Key search terms consisted of “biliary tract cancer” OR “cholangiocarcinoma” OR “gallbladder cancer” AND “MSI-high” OR “MSI-H” OR “dMMR.” Similar methods were implemented at the other sites. dMMR or MSI-H status was obtained from all patients with IHC/genomic reports, which were available for all patients. Clinical genomic reports were obtained where available including FoundationOne, TEMPUS, Guardant 360, Caris, Ambry Genetics, and MI-ONCOSEQ (FoundationOne; Foundation Medicine, Cambridge, MA; TEMPUS, Chicago, IL; Guardant Health, Redwood City, CA; Caris Life Sciences, Irving, TX; Ambry Genetics, Laguna Beach, CA; MI-ONCOSEQ, University of Michigan, Ann Arbor, MI).

### End Points

The end points were established in writing before data analysis. The primary study end point was rate of surgery and pathologic complete response (pCR) after ICI treatment.

The secondary end points were OS and TTD. OS was defined as time from initiation of first-line systemic therapy to death, and if still alive, patients were censored for OS at their last known alive date. TTD was defined as time from initiation of systemic treatment to cessation of treatment because of any reason, with patients still on treatment at the end of the reporting period being censored at the date of their most recent follow-up visit.

Objective response per clinician assessment was defined as either a partial or a complete response at any point during the administration of systemic therapy. Disease control was defined as having a complete response, partial response, or stable disease at any point during administration of therapy. ORR and disease control rate (DCR) were calculated among patients who initiated systemic therapy.

### Statistical Analysis

Statistical analysis was mainly descriptive. Continuous variables were described as medians with either IQR or range, and categorical variables were shown as frequency with percentages. The distribution of time to event end points was estimated using the Kaplan-Meier method. Analyses were performed using SAS (version 9.4; SAS institute, Cary, NC) and R (version 4.2.2). *P* values of <.05 were considered significant.

## RESULTS

### Patient Characteristics

**FIG A1.**
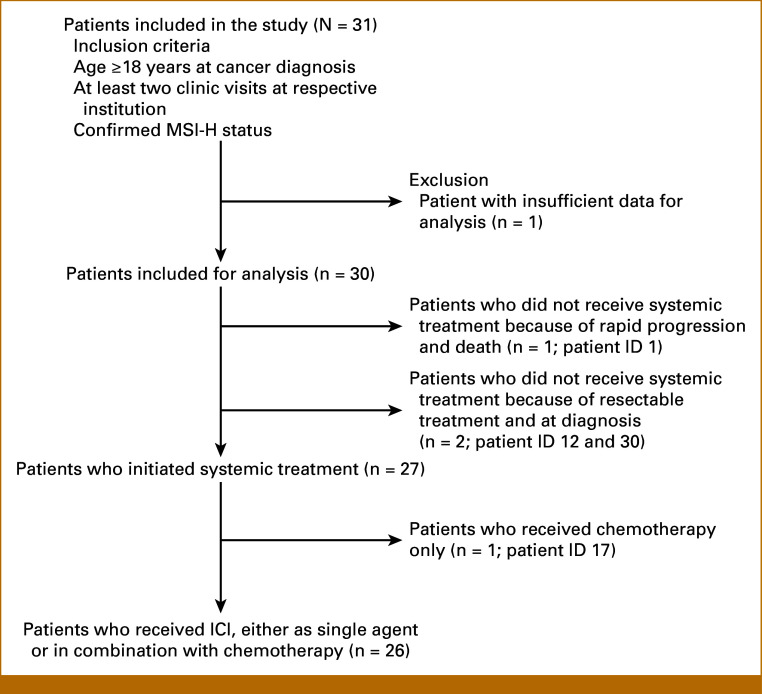
CONSORT diagram depicting criteria for patient selection. ICI, immune checkpoint inhibitor.

A total of 31 patients with MSI-H BTC were identified. Among these, one patient was excluded because of having insufficient follow-up for analysis, leaving a total of 30 patients for analysis (Appendix Fig A1).

The median age at diagnosis was 61.5 years (range, 26-86), with 28 (93.3%) patients being White and 17 (56.7%) being female. The median CA 19-9 was 35.5 U/mL (ranging 1.0-7478.0). At diagnosis, 19 (63.3%) patients had metastatic disease, 7 (23.3%) patients had locally advanced unresectable disease, and 4 (13.3%) patients had resectable disease at diagnosis. Potential underlying etiologies included Lynch syndrome (n = 8, 26.7%), gallstones (n = 3, 10%), primary sclerosing cholangitis (n = 2, 6.7%), and unknown (n = 17, 56.7%; Table 1).

**TABLE 1.** Baseline Characteristics (all patients)

Baseline Characteristic	Total (N = 30)
Age at diagnosis, years	
No. (missing)	30 (0)
Mean (SD)	61.6 (14.76)
Median	61.5
Range	26.0-86.0
IQR	56.0-72.0
Race, No. (%)	
White	28 (93.3%)
Black	1 (3.3%)
Unknown	1 (3.3%)
Sex, No. (%)	
Female	17 (56.7%)
Male	13 (43.3%)
CA19-9 at diagnosis	
No. (missing)	28 (2)
Mean (SD)	429.1 (1434.15)
Median	35.5
Range	1.0-7478.0
IQR	15.5-142.0
Stage at diagnosis, No. (%)	
Resectable	4 (13.3%)
Locally advanced	7 (23.3%)
Metastatic	19 (63.3%)
Stage at first-line treatment, No. (%)	
Locally advanced	6 (20.0%)
Metastatic	21 (70.0%)
Did not receive systemic treatment	3 (10.0%)
Tested for viral hepatitis, No. (%)	
No	13 (43.3%)
Yes	17 (56.7%)
Viral hepatitis status, No. (%)	
Positive	1 (3.3%)
Negative	16 (53.3%)
Unknown	13 (43.3%)
Etiology, No. (%)	
PSC	2 (6.7%)
Unknown	17 (56.7%)
Lynch syndrome	8 (26.7%)
Gallstones	3 (10.0%)

Abbreviation: SD, standard deviation.

### Treatment Details

Twenty-seven (90%) patients received systemic treatment. Reasons for not receiving systemic treatment include resection at diagnosis (n = 2, 6.7%) and rapid progression result in death (n = 1, 3.3%). Of the 27 patients who received first-line treatment, 12 (44.4%) received single-agent ICIs (n = 11 [40.7%] pembrolizumab, n = 1 [3.7%] nivolumab, treatment duration 1.3-41.8 months), 5 (18.5%) received ICI concurrent with chemotherapy (treatment duration 5.7-45.5 months), 9 (33.3%) received chemotherapy alone (treatment duration 0.7-7.6 months), and 1 (3.7%) participated in a clinical trial as their first-line therapy (treatment duration 8.5 months). Five (18.5%) of these patients remained on their first-line therapy at the last follow-up, and reasons for discontinuing first-line therapy included toxicity or functional decline (n = 7, 23.3%), disease progression (n = 6, 20%), transition to observation (n = 5, 16.7%), and undergoing surgery (n = 2, 6.7%; Table 2). Of the 27 patients, all 13 (48.1%) who came off first-line therapy because of progression or toxicity went on to receive second-line treatment. Among these 13 patients, 7 (53.8%) patients received single-agent ICIs (all pembrolizumab, treatment duration 2.5-27.1 months), 2 (15.4%) received ICI + chemotherapy (treatment duration 2.5-43.4 months), 2 (15.4%) received chemotherapy alone (treatment duration 0.9 months each), and 2 (15.4%) received treatment on a clinical trial (treatment duration 1-6.9 months). Six (46.2%) patients remained on treatment at last follow-up; among those who had discontinued second-line therapy, reasons for discontinuation included toxicity or functional decline (n = 4, 30.8%), disease progression (n = 1, 7.7%), transition to observation (n = 1, 7.7%), and undergoing surgery (n = 1, 7.7%).

**TABLE 2.** Treatment Details and Reasons for Discontinuation Among Patients Who Initiated First-Line Therapy

Treatment	Total (n = 27)
First-line treatment, No. (%)	
Clinical trial-Gem/Cis plus bintrafusp alfa	1 (3.7)
FOLFOX	1 (3.7)
Gem/Cis	6 (22.2)
Gem/Cis/Abraxane	1 (3.7)
Gem/Cis/Durva	4 (14.8)
Gem/Cis + pembrolizumab	1 (3.7)
Gemcitabine/oxaliplatin	1 (3.7)
Nivolumab	1 (3.7)
Pembrolizumab	11 (40.7)
Reason for discontinuation of first-line therapy, No. (%)	
Toxicity and/or functional decline	7 (25.9)
Disease progression	6 (22.2)
Transition to surveillance	5 (18.5)
Remain on treatment	5 (18.5)
Surgery	2 (7.4)
Unknown	1 (3.7)
Change of therapy	1 (3.7)
Second-line treatment, No. (%)	
Clinical trial	2 (15.4)
Durva + Gem/Cis	1 (7.7)
FOLFOX + pembrolizumab	1 (7.7)
Gem/abraxane	1 (7.7)
Gem/Cis	1 (7.7)
Pembrolizumab	7 (53.8)
Missing (patient who did not receive second-line treatment)	14
Reason for discontinuation of second-line therapy, No. (%)	
Remain on treatment	6 (46.2)
Toxicity and/or functional decline	4 (30.8)
Surgery	1 (7.7)
Disease progression	1 (7.7)
Transition to surveillance	1 (7.7)
Missing (patient who did not receive second-line treatment)	14

Abbreviations: Cis, Cisplatin; Durva, Durvalumab; Gem, Gemcitabine.

Only one (7.7%) of the patients who received second-line treatment continued on to receive third-line treatment (treatment duration 15 months). This patient received pembrolizumab and discontinued treatment because of disease progression.

Overall, 26 (96.3%) of the 27 patients who started first-line systemic treatment received an ICI agent—either as a single-agent ICI regimen or in combination with chemotherapy—17 (65.4%) received it as first-line therapy, and nine patients (34.6%) received it as second- or third-line therapy.

### End Points

#### Surgical Resection

**FIG A2.**
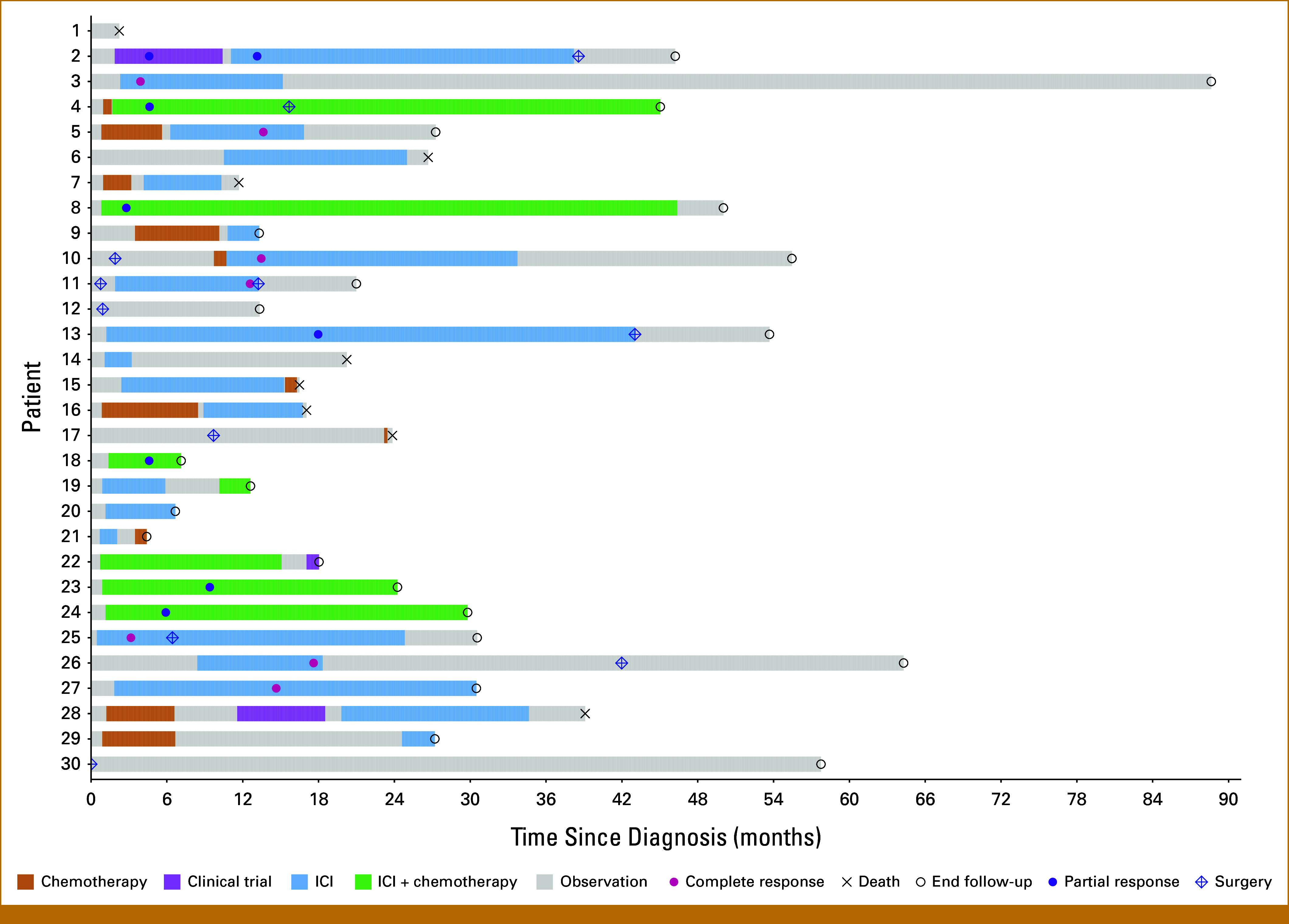
TTD in second-line treatment, patients who received second-line treatment. TTD, time to treatment discontinuation.

Six (23.1%) of the 26 patients who received ICI therapy (five with metastatic disease and one with locally advanced disease) underwent resection after ICI therapy, with no evidence of disease at the last follow-up. Postoperatively, 3(50%) patients had pCR on the resection specimen. These patients had also received a complete response on imaging before surgery. Of the 26 patients who received ICI, four patients (15.4%) achieved a partial response or complete response on imaging and remained recurrence-free at the end of the follow-up period. Individual patient timelines from diagnosis to last follow-up are depicted in Appendix Figure A2.

#### Overall Survival

**FIG 1.**
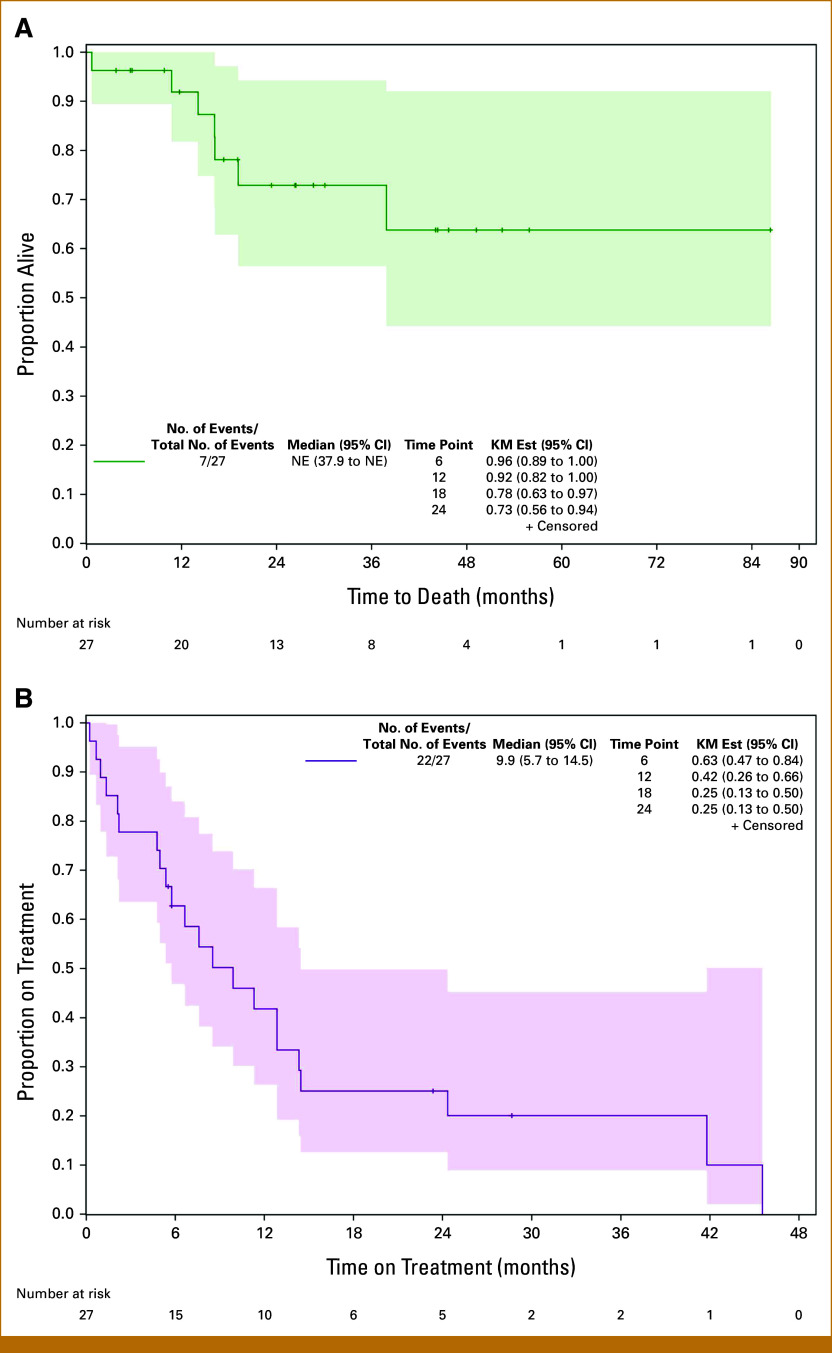
(A) OS from the start of first-line treatment, patients who received systemic treatment (n = 27). (B) TTD in first-line treatment, patients who received systemic treatment (n = 27). OS, overall survival; TTD, time to treatment discontinuation.

**FIG 2.**
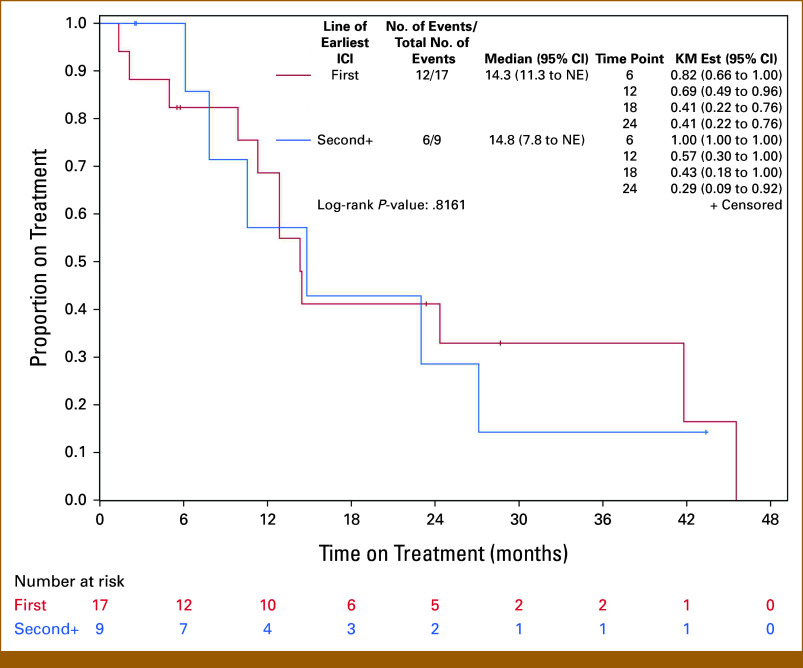
Kaplan-Meier curve depicting TTD from initiation to cessation of the earliest regimen that included an ICI, comparing those who received earliest ICI in first-line treatment versus second- or third-line treatment. ICI, immune checkpoint inhibitor; TTD, time to treatment discontinuation.

**FIG 3.**
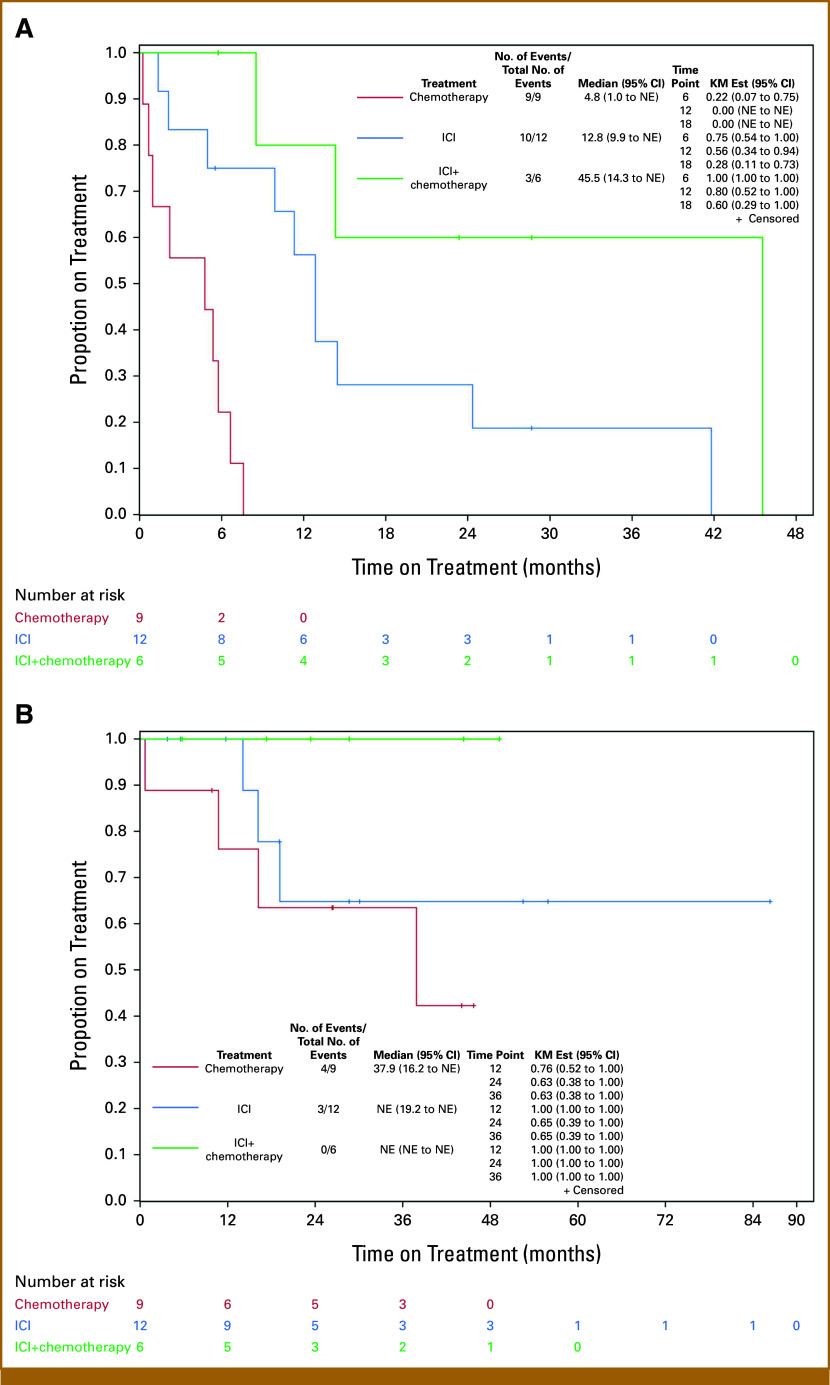
(A) Kaplan-Meier curve depicting TTD from initiation to cessation of first-line systemic treatment, grouped by type of systemic treatment. Patient who participated in the clinical trial received ICI concurrent with chemotherapy and is included in that group. (B) Kaplan-Meier curve depicting OS from initiation of first-line systemic treatment to death or last follow-up, grouped by the type of systemic treatment. Patients who participated in the clinical trial received ICI concurrent with chemotherapy and are included in that group. ICI, immune checkpoint inhibitor; OS, overall survival; TTD, time to treatment discontinuation.

**FIG A3.**
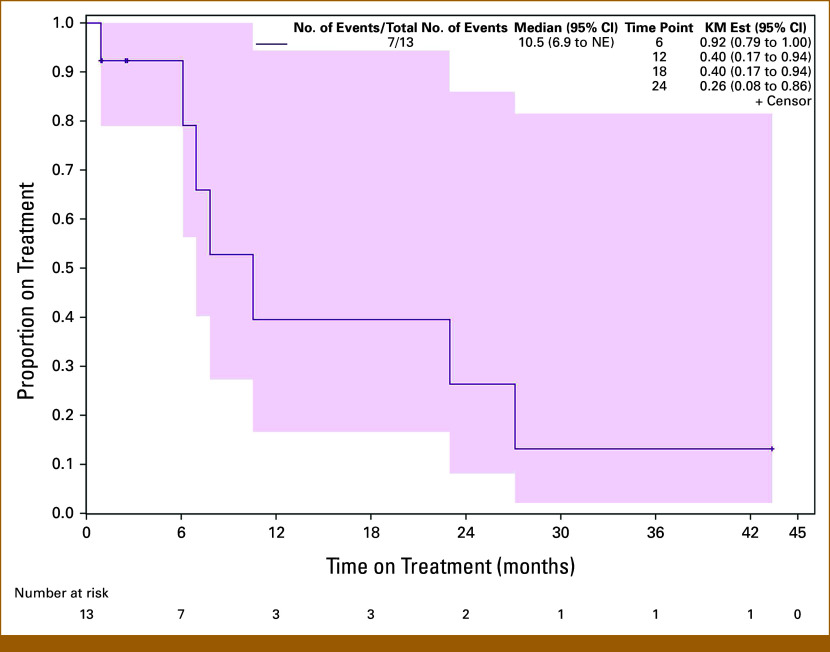
Swim plot depicting individual patient timelines from diagnosis (time 0) in months.

Among the 27 patients who received systemic treatment, the median (IQR) follow-up time was 28.6 (19.1, 45.7) months. The 2-year OS from the start of first-line treatment was 73% (95% CI, 56% to 94%), and the median TTD for patients on first-line treatment was 9.9 months (95% CI, 5.7 to 14.5 months; Figs 1A and 1B). The median TTD for second-line treatment was 10.5 months (95% CI, 6.9 to not estimable [NE]; Figure A3). Comparison of TTD of the earliest regimen versus second-line treatment and later, which included an ICI, revealed no significant difference: the median TTD (95% CI) for those who received earliest ICI in first-line treatment versus in second- or third-line treatment was 14.3 months (11.3-NE) versus 14.8 months (7.8-NE), respectively (*P* = .8161; Fig 2). However, analysis of first-line TTD by treatment type showed that ICI concurrent with chemotherapy (which includes the patient who participated in a clinical trial) had the longest median TTD of 45.5 months (95% CI, 14.3 to NE; n = 6), compared with 12.8 months (95% CI, 9.9 to NE; n = 12) for ICI alone and 4.8 months (95% CI, 1.0 to NE; n = 9) for chemotherapy alone. ICI concurrent with chemotherapy also had the highest 2-year OS rate at 1.00 (95% CI, 1.00 to 1.00), followed by ICI alone at 0.65 (0.39-1.00) and chemotherapy alone at 0.63 (95% CI, 0.38 to 1.00; Figs 3A and 3B).

Of the 27 patients who received first-line systemic treatment, 22 patients (81.5%) achieved disease control and 12 patients (44.4%) achieved objective response. When split by treatment type, patients who received ICI concurrent with chemotherapy had the highest rate of objective response of 83.3% compared with 58.3% for patients who received ICI alone and 0% for patients who received chemotherapy alone (Table 3). Subsequently, of the 13 patients who received second-line treatment, 8 (61.5%) achieved disease control and 4 (30.8%) achieved objective response.

**TABLE 3.** First-Line Objective Response and Disease Control Response by Treatment Group

Response Assessment	First-Line Chemotherapy Alone, n = 9 (%)	First-Line ICI Alone, n = 12 (%)	First-Line ICI Concurrent With Chemotherapy, n = 6 (%)	Total, n = 27 (%)
Objective response				
Yes	0 (0.0)	7 (58.3)	5 (83.3)	12 (44.4)
No	9 (100.0)	5 (41.7)	1 (16.7)	15 (55.6)
Disease control response				
Yes	6 (66.7)	10 (83.3)	6 (100.0)	22 (81.5)
No	3 (33.3)	2 (16.7)	0 (0.0)	5 (18.5)

Abbreviation: ICI, immune checkpoint inhibitor.

### Molecular Data

A total of 19 (63.3%) patients had complete next-generation sequencing (NGS) results, and 14 (46.6%) patients had IHC data available (Appendix Table A1). Tumor mutational burden was available for 18 (60%) patients. Median TMB was 26.5 mut/mb (O1, Q3: 18.9-39). The most common co-occurring mutations were *ARID1A*, *TP53*, and *PIK3CA*, seen in 11 (36.6%), 9 (30%), and 5 (16.6%) patients, respectively. Sixteen patients (53.3%) had completed germline testing, with eight patients (50%) found to have Lynch syndrome.

## DISCUSSION

In this multi-institutional study involving dMMR/MSI-H BTC, our findings show that patients who received systemic treatment had a 2-year OS of 73%. Furthermore, among patients who received an ICI as a part of their treatment profile, 23.1% converted to curative surgery and 11.5% achieved complete durable response not requiring surgery. These results underscore the vital importance of reflexive molecular testing up-front and timely administration of ICI in this highly aggressive cancer.

MSI-H is observed in a small subset of patients with BTC, approximately 1%-5%,^22-27^ and the advent of immunotherapy has led to improved outcomes in these patients with advanced disease. The KEYNOTE-158 study investigated the role of pembrolizumab in MSI-H patients with noncolorectal cancer, wherein the 2-year OS was observed to be 48.9% across 27 tumor types, inclusive of BTC.^16^ Our study found that the 2-year OS of patients who received systemic treatment was 73%, higher than the 2-year OS observed above, which is likely a reflection of the high rate of administration of ICI in the first-line and second-line setting in our population. This is in comparison with more than 33% of patients in KEYNOTE-158 having received three or more prior lines of therapy. Our results are also consistent with other prior studies.^28-30^ A study by Yang et al compared outcomes after administration of a PD-1 inhibitor and found that 16 patients with cholangiocarcinoma demonstrating MSI-H or deficient MMR tumors had a longer median OS (NE *v* 13.5 m, HR, 0.17 [95% CI, 0.06 to 0.46]; *P* = .001) and longer median PFS (NE *v* 4.0 m, HR, 0.14 [95% CI, 0.05 to 0.34]; *P* < .001) compared with microsatellite stable patients.^28^ Thus, advanced BTC patients with MSI-H stand to gain even greater response and benefits from early ICI treatments.

While ICI treatment in patients with advanced MSI-H BTC has demonstrated survival benefits, the role of neoadjuvant immunotherapy in early stage MSI-H BTC is not established. It has been postulated that response rates to ICI therapy are higher in early stage MSI-H solid tumors.^31^ A study by Ludford et al theorizes that this heterogeneity in response owes to immune evasion acquired by advanced tumors that enable metastatic dissemination. The study observed a radiographic response rate of 82% when using pembrolizumab in a neoadjuvant setting in patients with early-stage MSI-H solid tumors, compared with 44% and 34% in patients with metastatic MSI-H CRC and metastatic non-CRC solid tumors, respectively.^18,32^ Twenty-two patients with advanced CCA receiving pembrolizumab in a later-line setting in the KEYNOTE-158 study achieved an ORR of 40.9%, which, while higher than that observed in nonselective patients in the TOPAZ-1 study (ORR = 26.7%), is in line with that observed by Ludford et al in the metastatic setting.^2,16^ Our study comprised 90% of patients with advanced disease at the start of systemic therapy and demonstrated an ORR of 46.2% in first-line therapy and 30.8% in second-line therapy. Thus, ICI therapy might have higher clinical activity in patients if implemented earlier in their disease course.

The comparison of TTD of first-line versus second-line treatment and later with that of ICI treatment did not differ (14.3 months *v* 14.8 months, *P* = .081), which suggests that patients with MSI-H benefited from immunotherapy regardless of timing. However, timing may matter when functional status may change over time secondary to disease and thus may prohibit further therapy. Furthermore, TTD and objective response by treatment type may matter; patients who received chemotherapy alone in the first-line setting had a median TTD of 4.8 months and 0% response, patients who received ICI in the first-line setting had a median TTD of 12.8 months and a response of 58.3%, and patients who received ICI + chemotherapy in the first-line setting had a median TTD of 45.5 months and a response of 83.3%. However, this must be interpreted with caution given the small sample size, particularly the ICI + chemotherapy cohort.

Our study has several limitations. First, given the retrospective nature of this multicenter study, our results might have inherent selection bias. This study was performed at three high-volume tertiary centers, and patients may self-select to come and be evaluated/treated. While our results are consistent with those previously published, they should be interpreted with caution. Second, the small sample size of 30 patients restricts generalizability of the findings. However, to our knowledge, this is the largest study to date to address surgical conversion following ICI management and provides valuable real-world data in BTC. Third, while tissues and NGS were available, this was incomplete for all patients, thus limiting our ability to further assess TMB with response, tumor microenvironment, and the immune milieu in this heterogenous population.

In conclusion, this retrospective study on the largest cohort of patients with MSI-H BTC to date explores the rates of surgical conversion after ICI treatment. It reports promising outcomes in patients with unresectable and metastatic BTC treated with ICI, demonstrating that neoadjuvant ICI administration in patients can downstage unresectable disease and help achieve a pCR. Furthermore, ICI treatment can lead to complete responses long-term in a small subset of patients, thus allowing for an organ-preservation approach.

## APPENDIX

**TABLE A1.** MSI-H Status by IHC and NGS (where available)

Patient ID	dMMR by IHC	dMMR by NGS	Other Actionable Mutations	TMB (muts/mb)
1	NA	No specific mutation reported	ARID1A, KRAS	28
2	MLH1, PMS2	MLH1, MSH6	TSC2, TP53, ARID1A, MSH3	49.5
3	MLH1, PMS2	NA	NA	25
4	NA	MSH6	IDH1, PIK3CA	18.4
5	MLH1, PMS2	MLH1, PMS2	ATM, PIK3CA	30.0
6	NA	MLH1	FGFR3, PIK3CA, ARID1A, FGFR1, TP53, MSH3	19
7	MLH1, PMS2	MLH1	ARID1A, FGFR2, NF1	16
8	NA	No specific mutation reported	BRAF V600E, TP53	47
9	MLH1	NA	NA	NA
10	MSH2, MSH6	MLH1	BAP1, ARID1A, NF1, PBRM1	22
11	MSH2, MSH6	MSH6	ERBB2, CDK12, TP53, ARID1A, MSH3	38.9
12	MSH2, MSH6	MSH6	KRAS, BRIP1, ARID1A, MSH3	24.2
13	MLH1, PMS2	No specific mutation reported	NF1, TP53	Not calculated
14	MLH1, PMS2	No specific mutation reported	PBRM1, TP53, PIK3CA, PIK3R1, ARID1A, NF1, MSH3	18.9
15	MLH1, PMS2	No specific mutation reported	PBRM1, TP53, ARID1A, KRAS	8.4
16	NA	MSH2	TP53, ARID1A, PBRM1	8.4
17	NA	MLH1	ARID1A, RNF43, TP53, KDM6A, PBRM1	30.5
18	NA	MLH1, MSH2	FGFR3, NF1, PIK3CA, BRCA1, CDK12, ATM, PALB2, KRAS	380.47
19	NA	NA	NA	NA
20	MLH-1, PMS-2	MLH-1, PMS-2	NA	NA
21	NA	NA	NA	NA
22	NA	NA	NA	43
23	NA	NA	NA	NA
24	MLH1, PMS2	NA	NA	NA
25	NA	NA	NA	NA
26	NA	MSH2, MSH6	NA	NA
27	NA	NA	NA	NA
28	MSH2, MSH6	NA	NA	NA
29	NA	NA	NA	39
30	NA	MLH1, PMS2	NA	NA

Abbreviations: dMMR, deficient mismatch repair; IHC, immunohistochemistry; MSI, microsatellite instability; NA, not available; NGS, next-generation sequencing; TMB, tumor mutational burden.
